# Diagnostic performance of a urine-based ELISA assay for the screening of human schistosomiasis japonica: A comparative study

**DOI:** 10.3389/fmicb.2022.1051575

**Published:** 2022-11-14

**Authors:** Yi Mu, Kosala G. Weerakoon, Remigio M. Olveda, Allen G. Ross, Donald P. McManus, Pengfei Cai

**Affiliations:** ^1^Molecular Parasitology Laboratory, Infection and Inflammation Program, QIMR Berghofer Medical Research Institute, Brisbane, QLD, Australia; ^2^Department of Health, Research Institute for Tropical Medicine, Manila, Philippines; ^3^Research Institute for Rural Health, Charles Sturt University, Orange, NSW, Australia

**Keywords:** schistosomiasis, *Schistosoma japonicum*, diagnosis, urine, ELISA, droplet digital PCR, POC-CCA

## Abstract

The current study developed and evaluated the performance of a urine-based enzyme-linked immunosorbent assay (ELISA) for the screening of *Schistosoma japonicum* infection in a human cohort (*n* = 412) recruited from endemic areas, Northern Samar, the Philippines. The diagnostic performance of the urine ELISA assay was further compared with the Kato-Katz (KK) technique, serum-based ELISA assays, point-of-care circulating cathodic antigen (POC-CCA) urine cassette test, and droplet digital (dd)PCR assays performed on feces, serum, urine, and saliva samples, which were designated as F_ddPCR, SR_ddPCR, U_ddPCR, and SL_ddPCR, respectively. When urine samples concentrated 16× were assessed, the SjSAP4 + Sj23-LHD-ELISA (U) showed sensitivity/specificity values of 47.2/93.8% for the detection of *S. japonicum* infection in KK-positive individuals (n = 108). The prevalence of *S. japonicum* infection in the total cohort determined by the urine ELISA assay was 48.8%, which was lower than that obtained with the F_ddPCR (74.5%, *p* < 0.001), SR_ddPCR (67.2%, *p* < 0.001), and SjSAP4 + Sj23-LHD-ELISA (S) (66.0%, *p* < 0.001), but higher than that determined by the Sj23-LHD-ELISA (S) (24.5%, *p* < 0.001), POC-CCA assay (12.4%, *p* < 0.001), and SL_ddPCR (25.5%, *p* < 0.001). Using the other diagnostic tests as a reference, the urine ELISA assay showed a sensitivity between 47.2 and 56.9%, a specificity between 50.7 and 55.2%, and an accuracy between 49.3 and 53.4%. The concentrated urine SjSAP4 + Sj23-LHD-ELISA developed in the current study was more sensitive than both the KK test and POC-CCA assay, and showed a comparable level of diagnostic accuracy to that of the U_ddPCR. However, its diagnostic performance was less robust than that of the F_ddPCR, SR_ddPCR, and SjSAP4 + Sj23-LHD-ELISA (S) assays. Although they are convenient and involve a highly acceptable non-invasive procedure for clinical sample collection, the insufficient sensitivity of the three urine-based assays (the urine ELISA assay, the U_ddPCR test, and the POC-CCA assay) will limit their value for the routine screening of schistosomiasis japonica in the post mass drug administration (MDA) era, where low-intensity infections are predominant in many endemic areas.

## Introduction

One of the major neglected tropical diseases, schistosomiasis, caused by three main species of schistosomes (trematode blood flukes) infecting humans, *Schistosoma mansoni*, *S. haematobium*, and *S. japonicum*, affects more than 230 million people worldwide (McManus et al., 2018). The control of the disease relies predominantly on mass drug administration (MDA) programs with praziquantel; however, MDA on its own is insufficient to provide long-term sustainable control of the disease (Ross et al., 2015). Due to the COVID-19 pandemic, it has been reported that a total of 28.6 million fewer people were treated for schistosomiasis in 2020 than in 2019 with preventive treatment targeting school-aged children decreased from 66.8% in 2019 to 44.9% in 2020 (World Health Organization, 2021). Schistosomiasis outbreaks during the COVID-19 pandemic were also reported due to MDA program activities being postponed in 2020 across the endemic countries (Olamiju et al., 2022). The impact of the disruptions to schistosomiasis control programs due to COVID-19 thus needs to be closely monitored and evaluated. In this regard, the need for highly accurate, as well as cost-effective and convenient diagnostics has become a major priority.

In Asia, the major endemic foci of intestinal human schistosomiasis, caused by *S. japonicum* infection, are found in China, the Philippines, and small pockets of Indonesia (Ross et al., 2013). The transmission of the disease has decreased substantially in China due to extensive integrated control efforts (Gordon et al., 2021). In the Philippines, the infection remains endemic in 28 provinces across 12 geographical zones, with endemicity mostly located in the Central and Southern parts of the country (Olveda and Gray, 2019). Two and half million Filipinos are directly exposed to schistosomiasis, with approximately 12 million residing in endemic zones (Department of Health, 2018). As of 2019, it was reported that the national prevalence of schistosomiasis in the country was 4.0% (Belizario et al., 2022). However, this figure was derived from focal surveys and only one or two microscopy-based Kato-Katz (KK) slides were read for each individual, which may significantly underestimate the true burden of the disease (Olveda et al., 2016). Indeed, an integrated surveillance system has been recommended to help identify high-priority areas for targeted interventions, entailing the development of affordable and accurate diagnostic tools for rapid mapping and monitoring of schistosomiasis in the endemic zones in the Philippines (Belizario et al., 2022).

Currently, a variety of diagnostic methods are available for the detection of schistosome infection (Cavalcanti et al., 2013; Weerakoon et al., 2015). Parasitological-based diagnostics (e.g., the KK procedure for *S. japonicum* and *S. mansoni* detection, and the miracidium hatching technique) exhibit a considerable level of specificity but compromised sensitivities, particularly when used in endemic areas with reduced schistosome infection intensity (Cavalcanti et al., 2013). Improved coprological tests, such as the saline gradient method (Coelho et al., 2009), formalin-ethyl acetate sedimentation-digestion (FEA-SD) (Xu et al., 2012), and the Helmintex method (a procedure that isolates eggs from fecal samples with the use of paramagnetic particles in a magnetic field) (Lindholz et al., 2018), showed increased diagnostic sensitivity compared with the traditional techniques; on the down side, these procedures are usually labor-intensive, and have a lengthy processing time. Molecular methods based on polymerase chain reaction (PCR) technology, including real-time quantitative (q)PCR (Cnops et al., 2013; Gordon et al., 2015; Magalhaes et al., 2020; Mu et al., 2020; Halili et al., 2021), loop-mediated isothermal amplification (LAMP) (Gandasegui et al., 2015, 2018; Garcia-Bernalt Diego et al., 2021), and droplet digital (dd) PCR-based tests (Weerakoon et al., 2016, 2017a,b), are alternatives for schistosomiasis surveillance due to their exceptional diagnostic performance; however, these tests are expensive (e.g., the high price of DNA extraction, qPCR assay reagents, and equipment), and require experienced human resources, making their implementation in resource-limited areas challenging.

We recently developed serum-based IgG-ELISA assays targeting a pair of recombinant antigens or antigen combinations for diagnosis of *S. japonicum* in cohorts from endemic areas in the Philippines (Cai et al., 2017, 2019). The serum-based SjSAP4 + Sj23-LHD-ELISA assay [SjSAP4 + Sj23-LHD-ELISA (S)] exhibited a high level of diagnostic performance with 87.04% sensitivity and 96.67% specificity in testing 108 KK-positive subjects, the majority harboring low-intensity *S. japonicum* infections (Cai et al., 2017). The schistosomiasis prevalence determined by the SjSAP4 + Sj23-LHD-ELISA (S) was similar to that obtained by ddPCR assays performed on feces and serum samples, and was about 2.7-fold higher than that obtained with the KK procedure (Cai et al., 2019). However, serum-based diagnostic assays involve the invasive, onerous, and potentially hazardous of blood sampling (Archer et al., 2020). In contrast, urine-based diagnostic tests involve convenient and completely non-invasive urine sampling, with the limited chance of participants being exposed to biological risks. Previously, urine-based ELISA assays have been suggested as a possible alternative tool to diagnose a number of parasitic helminthiases such as echinococcosis, filariasis, opisthorchiasis, and schistosomiasis (Itoh et al., 2001, 2003; Sunita et al., 2007; Sawangsoda et al., 2012; Samad et al., 2013; Chirag et al., 2015; Eamudomkarn et al., 2018; Nagaoka et al., 2021; Chungkanchana et al., 2022). In this study, we aimed to validate a urine-based SjSAP4 + Sj23-LHD-ELISA assay for the diagnosis of *S. japonicum* infection in a human cohort recruited from areas in the Philippines moderately endemic for schistosomiasis japonica. The performance of the urine ELISA assay was further comprehensively compared with other diagnostic tests, including the KK procedure, serum-based ELISA assays (Cai et al., 2017), point-of-care circulating cathodic antigen (POC-CCA) urine cassette test (Cai et al., 2021), and ddPCR assays performed on feces, serum, urine, and saliva samples, which were designated as F_ddPCR, SR_ddPCR, U_ddPCR, and SL_ddPCR, respectively (Weerakoon et al., 2017b).

## Materials and methods

### Ethics statement

Human research ethical approval for the study was obtained from the Institutional Review Board (IRB) of the Research Institute for Tropical Medicine (RITM), Manila, the Philippines (IRB Number 2015-12), and the Human Research Ethics Committee (HREC), QIMR Berghofer Medical Research Institute (QIMRB), Brisbane, Australia (Ethics Approval: P524). Written consent was obtained from each study participant (for children under the age of 15 years, written consent was obtained from their legal guardians).

### Study cohort, sample collection, processing, and storage

The study recruited human subjects from 18 barangays moderately endemic for schistosomiasis japonica in the municipalities of Laoang and Palapag, Northern Samar, the Philippines, in 2015 (Cai et al., 2017, 2019; Weerakoon et al., 2017b). A variety of clinical samples (feces, serum, urine, and saliva) were collected from the participating subjects. For each participant, two fecal samples (10–15 g each) were sought on different days within a week for the Kato-Katz test. After fixing in 80% ethanol, the remainder of the first fecal sample (~10 g) was stored at 4°C. A blood sample (10 ml) was collected from each individual using a 10 ml serum silica vacutainer. The blood sample was allowed to clot at ambient temperature for 30 min. After centrifugation at 1,500× *g* for 10 min, the serum sample was then aliquoted and stored at 2–8°C. Around 40 ml of spot urine sample was collected in a 50 ml Falcon tube from each participant. Saliva (~2 ml) was collected into a 5 ml centrifuge tube using the passive drool method. All clinical samples were stored at 4°C and transported on wet ice to the RITM, where the samples were stored at −20°C. All samples were subsequently shipped to QIMRB, Brisbane, Australia, on dry ice for further analysis. Urine samples from healthy individuals from a non-endemic area in Australia were used as negative controls (*n* = 16).

### Parasitological detection

Kato-Katz (KK) analysis on the stool samples collected was performed at RITM as previously described (Gordon et al., 2015). Six thick smear slides per participant (three from each stool sample) were prepared and examined under a light microscope by experienced technicians. Infection intensity was recorded as the number of eggs per gram of feces (EPG). To ensure the accuracy of the KK test, 10% of the KK slides were randomly re-examined by an experienced microscopist.

### Urine ELISA

Urine samples were concentrated as previously described (Cai et al., 2021). Briefly, after thawing, urine samples were thoroughly mixed and centrifuged at 3,700× *g* for 10 min to remove urinary sediment. The supernatants were concentrated 20 times using 4 ml single-use 10 kDa Amicon Ultra filter units (Merck Millipore, Bayswater, VIC, Australia). The ELISA procedure was performed as described previously (Cai et al., 2017). MaxiSorp high protein-binding capacity 96-well ELISA plates (Nunc, Roskilde, Denmark) were coated with SjSAP4 and Sj23-LHD (0.5 μg/ml each in coating buffer; 100 μl/well) overnight at 4°C. Wells were blocked with PBST (Phosphate-buffered saline, pH 7.5 with 0.05% Tween-20) containing 1% (w/v) BSA for 1 h at 37°C. Urine samples 100 μl 10× concentrated urine samples (50 μl of concentrated urine samples plus 50 μl PBST containing 2% BSA) or 100 μl 16× concentrated urine samples (80 μl of concentrated urine samples plus 20 μl PBST containing 5% BSA) were added to the wells and the mixtures incubated for 1 h at 37°C. A mouse monoclonal anti-human IgG (Fc specific)-biotin antibody (Sigma-Aldrich Co, MO, USA) was then added as secondary antibody (1:20,000, 100 μl/well) for 1 h at 37°C. The plates were further incubated with Streptavidin-HRP (BD Pharmingen, CA, USA; 1:10,000, 100 μl/well) at 37°C for 0.5 h. Plates were washed with PBST for 5 times after each step. Colorimetric reactions were developed by adding 100 μl TMB substrate to each well and terminated after 5 min by adding 50 μl stop reagent (2 N H_2_SO_4_ per well). The ELISA plates were read at OD 450 nm with a POLARstar OPTIMA multidetection microplate reader (BMG LABTECH, VIC, Australia). Duplicate ELISA readings were undertaken. The cut-off value for a positive IgG response was set at 2.1 times the mean OD 450 nm value of urine samples from healthy controls.

### Comparative analysis using the KK, POC-CCA, ddPCR, and serum ELISA assays as reference

For ddPCR assays, DNA were extracted from the collected clinical samples (feces, serum, urine, and saliva) using the Maxwell 16 Instrument (Promega, Wisconsin; for fecal samples) or a ChemagicTM360 instrument (PerkinElmer Inc., Massachusetts; for serum, urine and saliva samples) (Weerakoon et al., 2017b). The purified DNA samples were analyzed on the QX200 ddPCR System (Bio-Rad) by amplifying an 82-bp fragment of the *S. japonicum* mitochondrial NADH I (nad1) gene (Weerakoon et al., 2017a,b). The ddPCRs performed on feces, serum, urine, and saliva were designated as F_ddPCR, SR_ddPCR, U_ddPCR, and SL_ddPCR, respectively. The ddPCR results are presented as the target gene copy number index (CNI) (Weerakoon et al., 2017b). For the POC-CCA assay, the detection of CCA in concentrated urine samples was performed using a commercially available POC-CCA urine cassette kit (Rapid Medical Diagnostics, Pretoria, South Africa) (Cai et al., 2021). The results of the POC-CCA assays were read after 20 min and transformed to a quantified value by introducing an R value, which was defined as the intensity of the test band divided by that of the control band within the same strip. In addition, three IgG-ELISA assays [Sj23-LHD-ELISA (S), SjSAP4-ELISA (S), and SjSAP4 + Sj23-LHD-ELISA (S)] were performed on the sera collected from the same cohort (Cai et al., 2017). Comparative analysis was undertaken on the results obtained for the urine ELISA assay and the other diagnostic methods (i.e., the KK, ddPCR, POC-CCA, and serum ELISA assays) used.

### Statistical analysis

All results were entered in a Microsoft Excel 2016 database. Statistical analysis was performed with GraphPad Prism Version 9 for windows (GraphPad Software, Inc., San Diego, CA, USA). All the data are presented as the mean ± standard error (SE). For the ELISA, the urine IgG responses between the different subsets of the cohort and healthy controls were analyzed by the Mann–Whitney *U*-test. Diagnostic performance was evaluated by receiver operating characteristic (ROC) curve analysis. The area under the ROC curve (AUC) was calculated to assess the diagnostic performance of the SjSAP4 + Sj23-LHD-ELISA (U) assay in the different subsets of the cohort. Differences in the positive rates and infection prevalences determined by the urine ELISA assay and the other diagnostic approaches (the ddPCR, POC-CCA, and serum ELISA assays) were tested using McNemar’s test across the different infection intensity groups. Using the different diagnostics methods (the KK, ddPCR, POC-CCA, and serum ELISA assays) as reference, sensitivity, specificity, positive predictive value (PPV), negative predictive value (NPV), and accuracy were analyzed for the urine ELISA assay. Agreement between the urine ELISA assay and the other tests was determined using the Kappa statistic.^1^ The strength of agreement was measured by the scale according to the κ value, with the scores divided into: <0, no agreement; 0.00–0.20 slight agreement; 0.21–0.40 fair agreement; 0.41–0.60 moderate agreement; 0.61–0.80 substantial agreement; and 0.81–1.00 perfect agreement (Landis and Koch, 1977).

## Results

### Study population and schistosome fecal egg burdens

A total of 412 participants were recruited from 18 barangays in a schistosomiasis-endemic rural area, Northern Samar, the Philippines. Among them, 108 (26.2%) participants were *S. japonicum* egg-positive determined by six smear slides from two stool samples (three slides each) with a mean EPG of 17.6. Of these KK-positives, the majority (*n* = 104; 96.1%) had a light-intensity infection (EPG < 100) (Cai et al., 2021). Sixteen subjects were recruited from a non-endemic area in Australia as controls.

### Optimization of loading volume of concentrated urine samples for ELISA assay

**Figure 1:**
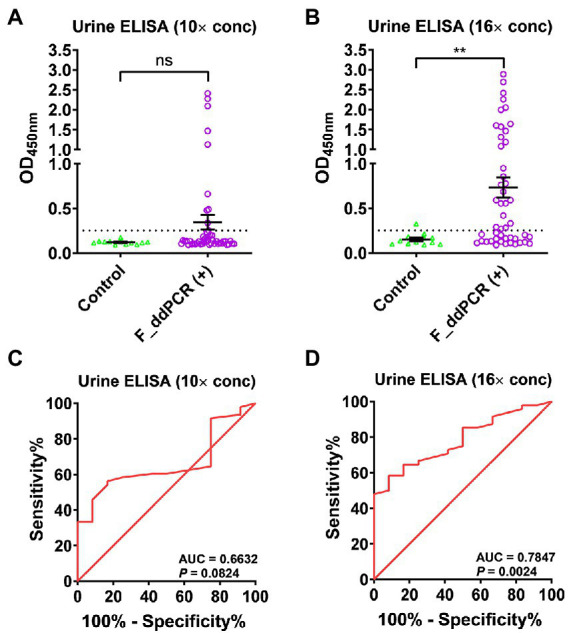
Determination of the optimal loading volume of concentrated (20×) urine samples for the SjSAP4 + Sj23-LHD-ELISA assay. **(A,B)** Scatter plots showing the IgG responses in 50 **(A)** and 80 μl **(B)** urine sample, respectively, to the *S. japonicum* antigen combination SjSAP4 + Sj23-LHD. Healthy controls (*n* = 12), F_ddPCR-positives (*n* = 48). *p* values were determined using the Mann–Whitney *U*-test. ns, no significant difference; **p < 0.01. **(C,D)** Receiver operating characteristic curve (ROC) analysis was performed to evaluate the capability of the urine ELISA in discriminating the healthy control group (*n* = 12) and F_ddPCR-positives (*n* = 48). Fifty μl **(C)** and 80 μl **(D)** concentrated (20×) urine samples were loaded on the plate during the ELISA assay, respectively.

Urine samples were concentrated 20 times using 10 kDa Amicon Ultra filter units. We then optimized the loading volume of concentrated (20×) urine samples in the SjSAP4 + Sj23-LHD-ELISA (U) assay. By employing 48 F_ddPCR-positive subjects and 12 healthy controls, 50 or 80 μl concentrated (20×) urine samples (i.e., the final urine concentration is 10× and 16×, respectively) were tested in the SjSAP4 + Sj23-LHD-ELISA (U) assay. No significant difference in the IgG levels was observed between individuals in the control group and the F_ddPCR-positives at a urine concentration of 10× (Figure 1A), whereas the OD values of the SjSAP4 + Sj23-LHD-ELISA (U) assay were significantly higher in the F_ddPCR-positives than those of the healthy control group (*p* = 0.0018) at a urine concentration of 16× (Figure 1B). The ROC analysis indicated that the SjSAP4 + Sj23-LHD-ELISA (U) was unable to discriminate the F_ddPCR-positives from the healthy controls (*p* = 0.0824) when the urine concentration was at 10× (Figure 1C). The ROC analysis for discriminating F_ddPCR-positives from the healthy controls showed that the AUC level for the urine ELISA assay was 0.7847 (*p* = 0.0024) when 16× concentrated urine samples were tested (Figure 1D).

### Diagnostic performance of the SjSAP4 + Sj23-LHD-ELISA (U) test using different subsets of diagnosed positives with other tests as a reference

**Figure 2:**
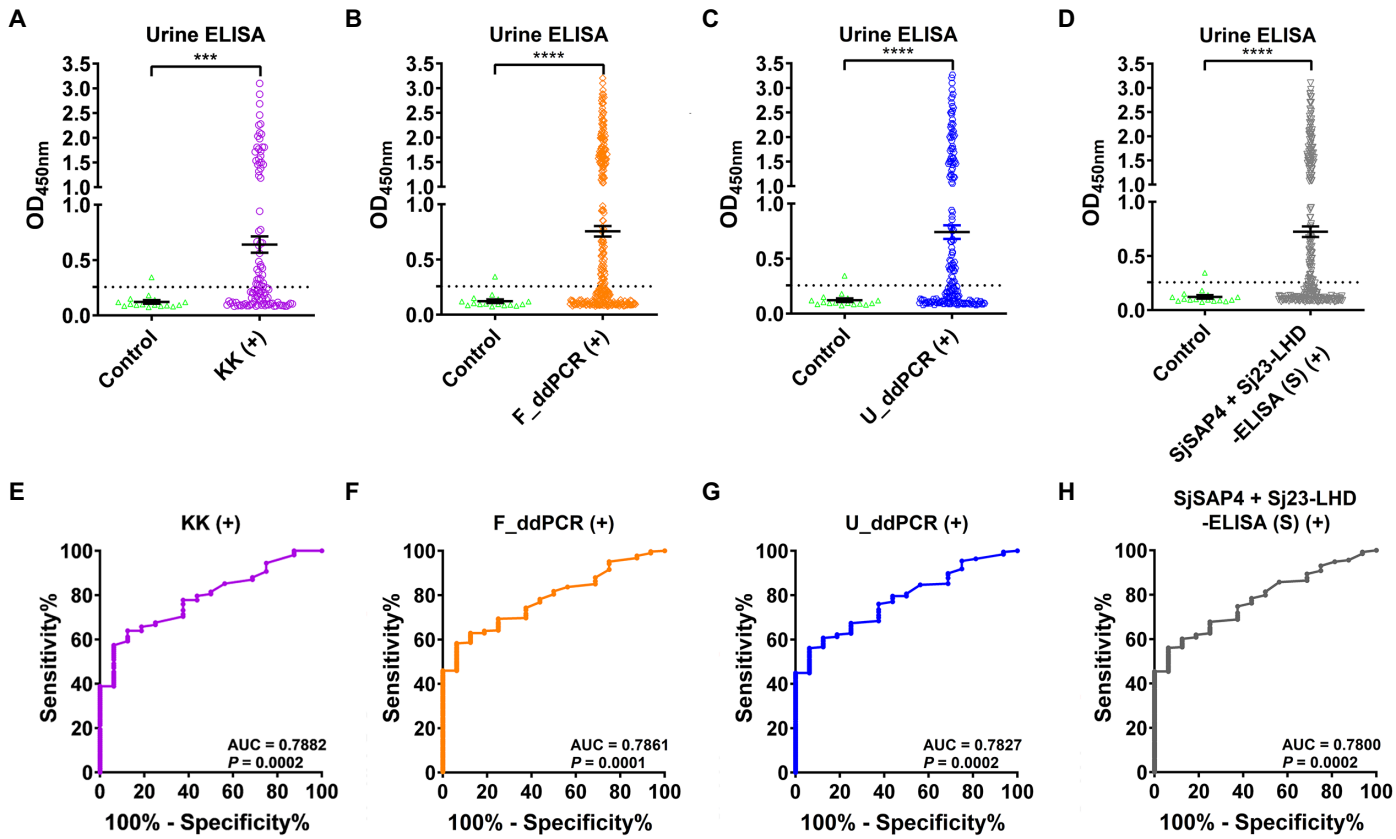
Urinary IgG levels against the antigen combination SjSAP4 + Sj23-LHD in different subgroups. **(A–D)** Scatter plots showing the urine IgG responses to SjSAP4 + Sj23-LHD in the KK-positives (*n* = 108; **A**), F_ddPCR-positives (*n* = 307; **B**), U_ddPCR-positives (*n* = 196; **C**) and serum SjSAP4 + Sj23-LHD-ELISA-positives (*n* = 272; **D**), respectively, for the diagnosis of schistosomiasis japonica. Urine samples from 16 healthy individuals were used as controls. *p* values were determined using the Mann–Whitney *U*-test. ***p < 0.001; ****p < 0.0001. **(E–H)** ROC analysis was performed to evaluate the capability of the urine ELISA in discriminating the healthy control group and different subsets of the cohorts [KK-positives **(E)**, F_ddPCR-positives **(F)**, U_ddPCR-positives **(G)** and serum SjSAP4 + Sj23-LHD-ELISA (S)-positives **(H)**, respectively].

We then tested the SjSAP4 + Sj23-LHD-ELISA (U) assay on all 16× concentrated urine samples of the endemic human cohort (*n* = 412) and healthy controls (*n* = 16). IgG levels of the urine ELISA assay were significantly higher in the KK-, F_ddPCR-, U_ddPCR-, and serum SjSAP4 + Sj23-LHD-ELISA-positive groups (*n* = 108, 307, 196, and 272, respectively) than those of the healthy control group (Figures 2A–D). The urine ELISA assay showed a diagnostic sensitivity of 47.2, 50.2, 49.0, and 50.0%, respectively, for the diagnosis of *S. japonicum* infection in the above-mentioned subgroups, with a specificity of 93.8%. The ROC analysis for discriminating the KK, F_ddPCR, U_ddPCR, and serum SjSAP4 + Sj23-LHD-ELISA-positive subjects from the healthy controls showed that the AUC level for the urine ELISA assay was 0.7882, 0.7861, 0.7827 and 0.7800, respectively (*p* = 0.0002, 0.0001, 0.0002 and 0.0002, respectively; Figures 2E–H).

### Positivity rate and prevalence analysis

In the KK-moderate infection group (EPG: 100–399), the SjSAP4 + Sj23-LHD-ELISA (U) assay showed a 75% positivity rate, while the other diagnostic tests had a 100% positivity rate. In the subgroup with a low egg burden (EPG: 10–99, *n* = 26), the positivity rate was significantly higher when determined by the F_ddPCR (100%, *p* = 0.0015), SR_ddPCR (100%, *p* = 0.0015), U_ddPCR (88.5%, *p* = 0.0265), the SjSAP4-ELISA (S) (92.3%, *p* = 0.0094), and the SjSAP4 + Sj23-LHD-ELISA (S) (92.3%, *p* = 0.0094) compared with the SjSAP4 + Sj23-LHD-ELISA (U) assay (53.8%). There was no difference when the positivity rate determined by the SL_ddPCR, POC_CCA, and the SjSAP4-ELISA (S) were compared with the urine ELISA assay. In all subjects with an extremely low egg burden (EPG: 1–9, n = 78), the positivity rate determined by the urine ELISA assay (43.6%) was significantly lower than that obtained with the F_ddPCR (97.4%, *p* < 0.0001), the SR_ddPCR (92.3%, *p* < 0.0001), the SjSAP4-ELISA (S) (80.8%, *p* < 0.0001), and the SjSAP4 + Sj23-LHD-ELISA (S) (84.6%, *p* < 0.0001), but higher than those determined by the SL_ddPCR (24.4%, *p* = 0.0180) and the POC-CCA test (16.7%, *p* = 0.0007). In all KK-negatives (EPG: 0, n = 304), the positivity rate determined by the SjSAP4 + Sj23-LHD-ELISA (U) test (49.3%) was significantly lower than that obtained with the F_ddPCR (66.1%, *p* < 0.0001), and the SjSAP4 + Sj23-LHD-ELISA (S) (58.6%, *p* = 0.0265), but higher than that determined by the SL_ddPCR (20.4%, *p* < 0.0001), the POC-CCA test (6.3%, *p* < 0.0001), and the Sj23-LHD-ELISA (S) (18.1%, *p* < 0.0001; Table 1). Among the total cohort participants (n = 412), the prevalence determined by the urine ELISA assay (48.8) was significantly lower than that obtained with the F_ddPCR, SR_ddPCR, SjSAP4-ELISA (S), and the SjSAP4 + Sj23-LHD-ELISA (S) assays (*p* < 0.0001 in all comparisons), but higher than that resulting from the SL_ddPCR, POC-CCA, and Sj23-LHD-ELISA (S) assays (*p* < 0.0001 in all comparisons); no difference was evident in prevalence between the urine ELISA assay and that obtained with the U_ddPCR assay (*p* > 0.05; Table 1).

**Table 1:** Comparison of positivity rates in different subgroups and prevalence in the whole cohort determined by the urine ELISA assay with those obtained with other diagnostic tests.

Diagnostic test	KK (+) moderate	KK (+) light^§^				KK (−)		Whole cohort	
	(EPG: 100–399)	(EPG: 10–99)		(EPG: 1–9)		(EPG: 0)		(EPG: 0–399)	
	% positive	% positive	*p* *	% positive	*p* *	% positive	*p* *	% prevalence	*p* *
SjSAP4 + Sj23-LHD-ELISA (U)^†^	75 (3/4)	53.8 (14/26)		43.6 (34/78)		49.3 (150/304)		48.8 (201/412)	
F_ddPCR	100 (4/4)	100 (26/26)	0.0015	97.4 (76/78)	<0.0001	66.1 (201/304)	<0.0001	74.5 (307/412)	<0.0001
SR_ddPCR	100 (4/4)	100 (26/26)	0.0015	92.3 (72/78)	<0.0001	57.6 (175/304)	>0.05	67.2 (277/412)	<0.0001
U_ddPCR	100 (4/4)	88.5 (23/26)	0.0265	47.4 (37/78)	>0.05	43.4 (132/304)	>0.05	47.6 (196/412)	>0.05
SL_ddPCR	100 (4/4)	76.9 (20/26)	>0.05	24.4 (19/78)	0.0180	20.4 (62/304)	<0.0001	25.5 (105/412)	<0.0001
POC-CCA^#^	100 (4/4)	57.7 (15/26)	>0.05	16.7 (13/78)	0.0007	6.3 (19/304)	<0.0001	12.4 (51/412)	<0.0001
Sj23-LHD-ELISA (S)^††^	100 (4/4)	53.8 (14/26)	>0.05	35.9 (28/78)	>0.05	18.1 (55/304)	<0.0001	24.5 (101/412)	<0.0001
SjSAP4-ELISA (S)^††^	100 (4/4)	92.3 (24/26)	0.0094	80.8 (63/78)	<0.0001	56.3 (171/304)	>0.05	65.5 (262/412)	<0.0001
SjSAP4 + Sj23-LHD-ELISA (S)^††^	100 (4/4)	92.3 (24/26)	0.0094	84.6 (66/78)	<0.0001	58.6 (178/304)	0.0265	66.0 (272/412)	<0.0001

§ Individuals with a light infection were categorised into two subgroups with EPGs of 10–99 and 1–9.

* *p* values were determined by McNemar’s test.

# *R* cut-off value: 0.1344 (Cai et al., 2021).

† OD 450 cut-off value for the urine ELISA assay: SjSAP4 + Sj23-LHD-ELISA (U), 0.2570.

†† OD 450 cut-off values for the serum ELISA assays: Sj23-LHD-ELISA (S), 0.2185; SjSAP4-ELISA (S), 0.1832; SjSAP4 + Sj23-LHD-ELISA (S), 0.2003 (Cai et al., 2017).

### A comparison of the performance of the SjSAP4 + Sj23-LHD-ELISA (U) assay with the other diagnostic tests in detecting *Schistosoma japonicum* infection

We determined the sensitivity, specificity, PPV, NPV, and accuracy of the SjSAP4 + Sj23-LHD-ELISA (U) using the KK, F_ddPCR, SR_ddPCR, U_ddPCR, SL_ddPCR, POC-CCA assay, Sj23-LHD-ELISA (S), SjSAP4-ELISA (S), and SjSAP4 + Sj23-LHD-ELISA (S), respectively, as the reference tests (Table 2). The SjSAP4 + Sj23-LHD-ELISA (U) had the highest sensitivity (56.9%), using the POC-CCA assay as the reference, followed by using the Sj23-LHD-ELISA (S) as the reference, showing a sensitivity of 54.5%. The urine ELISA assay showed an accuracy of about 50% when using other diagnostic methods as references, with the highest accuracy of 53.4% using the Sj23-LHD-ELISA (S) as the reference. A similar but moderate specificity (50.7–55.2%) was observed by comparing the SjSAP4 + Sj23-LHD-ELISA (U) assay with the other diagnostic tests. The Kappa statistics analysis indicated that the urine ELISA assay showed a slight concordance with the SjSAP4 + Sj23-LHD-ELISA (S), Sj23-LHD-ELISA (S), POC-CCA, and F_ddPCR assays (*κ* < 0.2), but no agreement with the other tests (*κ* < 0).

**Table 2:** Diagnostic performance of the urine ELISA assay using different tests as reference.

SjSAP4 + Sj23-LHD-ELISA (U)^†^	Reference test		% sensitivity (95% CI)	% specificity (95% CI)	% PPV (95% CI)	% NPV (95% CI)	% accuracy (95% CI)	Kappa index (95% CI)
	**+**	**−**						
	KK							
**+**	51	150	47.2 (38.1–56.6)	50.7 (45.1–56.2)	25.4 (19.9–31.8)	73.0 (66.6–78.5)	49.8 (45.0–54.8)	−0.017 (−0.102–0.069)
−	57	154												
	F_ddPCR							
**+**	154	47	50.2 (44.6–55.7)	55.2 (45.7–64.4)	76.6 (70.3–81.9)	27.5 (21.9–33.9)	51.5 (46.6–56.3)	0.041 (−0.042–0.124)
−	153	58						
	SR_ddPCR							
**+**	135	66	48.7 (42.9–54.6)	51.1 (42.8–59.4)	67.2 (60.4–73.3)	32.7 (26.7–39.3)	49.5 (44.7–54.3)	−0.001 (−0.091–0.089)
−	142	69						
	U_ddPCR							
**+**	96	105	49.0 (42.1–56.0)	51.4 (44.8–58.0)	47.8 (41.0–54.6)	52.6 (45.9–59.2)	50.2 (45.4–55.1)	0.004 (−0.093–0.100)
−	100	111						
	SL_ddPCR							
**+**	52	149	49.5 (40.2–58.9)	51.5 (45.9–57.0)	25.9 (20.3–32.3)	74.9 (68.6–80.3)	51.0 (46.2–55.8)	0.008 (−0.078–0.093)
−	53	158						
	POC-CCA^#^							
−	29	172	56.9 (43.3–69.5)	52.4 (47.2–57.5)	14.4 (10.2–20.0)	89.6 (84.7–93.0)	52.9 (48.1–57.7)	0.041 (−0.024–0.106)
**+**	22	189						
	Sj23-LHD-ELISA (S)^††^							
**+**	55	146	54.5 (44.8–63.8)	53.1 (47.5–58.5)	27.4 (21.7–33.9)	78.2 (72.2–83.2)	53.4 (48.6–58.2)	0.056 (−0.028–0.140)
−	46	165						
	SjSAP4-ELISA (S)^††^							
**+**	127	74	48.5 (42.5–54.5)	50.7 (42.8–58.6)	63.2 (56.3–69.6)	36.0 (29.8–42.7)	49.3 (44.5–54.1)	−0.008 (−0.100–0.084)
−	135	76						
	SjSAP4 + Sj23-LHD-ELISA (S)^††^							
**+**	136	65	50.0 (44.1–55.9)	53.6 (45.3–61.6)	67.7 (60.9–73.7)	35.6 (29.4–42.2)	51.2 (46.4–56.0)	0.032 (−0.063–0.118)
−	136	75						

# *R* cut-off value: 0.1344 (Cai et al., 2021).

† OD450 cut-off value for the urine ELISA assay: SjSAP4 + Sj23-LHD-ELISA (U), 0.2570.

†† OD450 cut-off values for the serum-based ELISA assays: Sj23-LHD-ELISA (S), 0.2185; SjSAP4-ELISA (S), 0.1832; SjSAP4 + Sj23-LHD-ELISA (S), 0.2003 (Cai et al., 2017).

### Schistosomiasis japonica prevalence analysis

**Figure 3:**
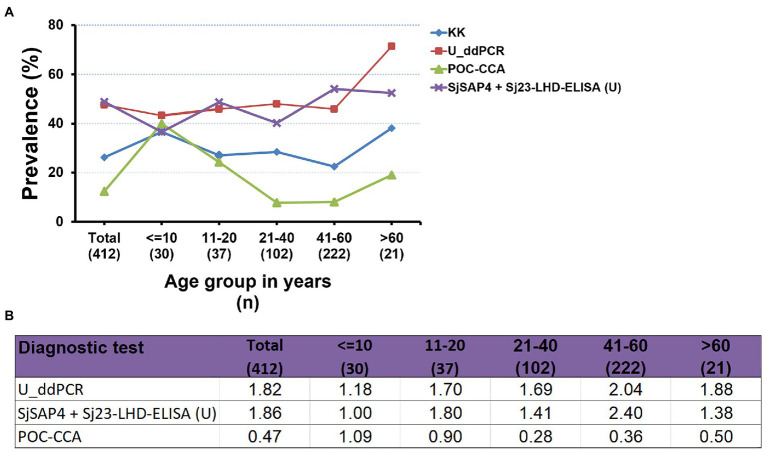
Comparison of the prevalence of *S. japonicum* infection determined by the different diagnostic tests. **(A)** The prevalence of schistosomiasis japonica determined by the KK procedure and three urine-based diagnostic tests [the U_ddPCR, SjSAP4 + Sj23-LHD-ELISA (U) and the POC-CCA assay] for the total human cohort by different age groups. **(B)** Fold changes in the prevalence of *S. japonicum* infection obtained with the U_ddPCR, the urine ELISA and the POC-CCA assay vs. the KK examined for the total cohort by each age group.

The prevalence of *S. japonicum* infection in the total human cohort and different age groups determined by the KK procedure and three urine-based tests, i.e., the U_ddPCR, the SjSAP4 + Sj23-LHD-ELISA (U) and the POC-CCA assay, is shown in Figure 3A. In the total cohort, the prevalence of schistosomiasis determined by the urine ELISA assay was similar to that determined by the U_ddPCR, and was about 1.8 times higher than obtained with the KK method, while the prevalence of schistosomiasis determined by the POC-CCA cassette test was about half of that obtained with the KK method (Figure 3A). The prevalence determined for each age group using the urine ELISA assay and the U_ddPCR test was between 1.0 and 2.4 times of that obtained with the KK method, while the prevalence determined by the immunochromatographic POC-CCA assay was between 0.24 and 1.09 times of that obtained with the KK method (Figure 3B).

## Discussion

Due to the relative ease and the non-invasive nature of urine collection, as well as the minimal technical and ethical issues involved in the procedure, urine represents an accessible clinical sample that can be readily collected from patients or healthy volunteers for diagnostic testing (Gao, 2013; Archer et al., 2020). While urine samples have been used widely for the diagnosis of African schistosomiasis (Tchuem Tchuente et al., 2012; Coulibaly et al., 2013; Casacuberta et al., 2016; de Dood et al., 2018; Mewamba et al., 2021; Mohammed et al., 2022), few studies have reported the utility of urine for the diagnostic testing of schistosomiasis japonica (Itoh et al., 2003; Weerakoon et al., 2017b; Cai et al., 2021). In the field setting, this is convenient where only a single urine sample is required whereas a minimum of two stool samples are required for the KK procedure. In addition, participants can personally collect a urine sample following simple instructions, without the need for personnel trained in venipuncture, and this can reduce the potential risk of individuals contracting and spreading other diseases such as COVID-19. We previously reported that a serum-based IgG-ELISA assay targeting the antigen combination SjSAP4 + Sj23-LHD was a promising diagnostic assay for the surveillance of *S. japonicum* infection (Cai et al., 2017, 2019). Here, we aimed to validate the diagnostic capacity of the SjSAP4 + Sj23-LHD-ELISA assay for the screening of *S. japonicum* infection by testing specific IgG antibodies in urine samples collected from a human cohort living in schistosomiasis-endemic areas of the Philippines.

Using the WHO criteria for KK-based EPG grading, the human cohort utilized in this study was characterized as having a moderate *S. japonicum* prevalence and a low infection intensity (Cai et al., 2019). Hence, there was a high possibility that there could have been even lower intensity infections in the community cohort, which would have been undetected by the KK procedure due to its inherent limited sensitivity. The present study confirmed this premise by demonstrating a low sensitivity of the KK test compared with the urine ELISA assay, i.e., the KK method exhibited 35% sensitivity (Weerakoon et al., 2017b) compared with 50.2% sensitivity of the urine ELISA procedure (Table 2), with both using the high performance F_ddPCR assay as reference test. We investigated the diagnostic performance of the urine ELISA assay against other molecular and serological methods using clinical samples collected from the well-defined cohort from the Philippines (Cai et al., 2017, 2019, 2021; Weerakoon et al., 2017b). While the F_ddPCR, SR_ddPCR, SjSAP4 + Sj23-LHD-ELISA (S), and SjSAP4-ELISA (S) recorded higher prevalence than the urine ELISA assay, the latter outperformed the SL_ddPCR, POC-CCA, and Sj23-LHD-ELISA (S) assays in the determination of schistosomiasis prevalence in the target human cohort (Table 1).

Previously, Itoh et al. (2003) developed an ELISA to detect IgG levels against *S. japonicum* soluble egg antigens (SEA) in un-concentrated urine samples collected from participants in a schistosomiasis japonica endemic area adjacent to the Dongting Lake, Hunan Province, China, in 1995 and 1996, showing that within 129 serum ELISA-positive individuals, 112 (86.8%) tested positive with the urine ELISA. When using the SjSAP4 + Sj23-LHD-ELISA (S) as reference, the sensitivity of the urine ELISA we obtained here was 50% (Table 2); the low sensitivity of the SjSAP4 + Sj23-LHD-ELISA (U) assay reflects the relatively low specific IgG concentration in the urine samples, as most of the cohort KK-positive individuals harbored an EPG less than 10 (Cai et al., 2021). For the SjSAP4 + Sj23-LHD-ELISA (S) assay, the optimized serum dilution was 1:250 (Cai et al., 2017). The concentration of antibodies in urine is only about 1/4,000 to 10,000 of that in serum (Nagaoka et al., 2021). Previously, it had been shown that the sensitivity of a urine-based ELISA was improved in the diagnosis of strongyloidiasis by adding a concentration protocol of urine samples (Chungkanchana et al., 2022). In addition, the diagnostic accuracy of the POC-CCA methodology was significantly increased by introducing a phase of urine concentration in two Brazilian *S. mansoni* endemic areas (Grenfell et al., 2018). In the present study, the SjSAP4 + Sj23-LHD-ELISA (U) assay exhibited an improved performance when 16× concentrated urine samples were tested compared with 10× concentrated samples. Nevertheless, the sensitivity of the SjSAP4 + Sj23-LHD-ELISA (U) assay was still relatively lower than that obtained with the SjSAP4 + Sj23-LHD-ELISA (S) assay [50.2% vs. 76.5% (Cai et al., 2019) using the F_ddPCR as reference test], which is a common phenomenon observed when comparing the sensitivity of serum and urine ELISA assays against the same antigen/s (Pearson et al., 2021).

Overall, the SjSAP4 + Sj23-LHD-ELISA (U) assay developed in the current study exhibited a diagnostic performance commensurate with that of the U_ddPCR. Although the U_ddPCR showed a higher positivity rate than that of the urine ELISA assay (88.5% vs. 53.8%, *p* = 0.0265) in the group of individuals with an EPG 10–99, there was no difference in the positivity rate for the SjSAP4 + Sj23-LHD-ELISA (U) and U_ddPCR in the group of individuals with an EPG of 1–9 (43.6% vs. 47.4%, *p* > 0.05) and in KK-negatives (49.3% vs. 43.4%, *p* > 0.05), respectively (Table 2); this resulted in a similar schistosomiasis prevalence determined by the urine ELISA assay and the U_ddPCR assay (48.8% vs. 47.6%, *p* > 0.05), i.e., about 1.8-fold higher than that obtained with the KK procedure (26.2%). When using the accurate F_ddPCR as reference, the urine ELISA assay and the U_ddPCR test showed a similar sensitivity (50.2% vs. 49%), specificity (55.2% vs. 57%; Table 2; Weerakoon et al., 2017b), accuracy (51.5% vs. 51.2%), and agreement (0.041 vs. 0.047; Table 2; Supplementary Table S1).

The POC-CCA assay has been evaluated extensively for the rapid diagnosis of schistosomiasis, particularly for *S*. *mansoni* infection. It has been shown in schistosomiasis mansoni-endemic areas with a prevalence less than 50% by KK, the prevalence determined by the POC-CCA assay was between 1.5- and 6-fold higher than that obtained with the KK procedure (Kittur et al., 2016). We previously detected the presence of *Schistosoma* circulating cathodic antigen (CCA) in concentrated urine samples (the same samples as used in the current study) by the commercial available POC-CCA strips, which showed a very limited sensitivity (29.6% and 14.3% using the KK and the F_ddPCR as reference test, respectively) in detecting *S. japonicum* infection (Cai et al., 2021). In contrast, the urine ELISA assay had a sensitivity of 47.2% and 50.2% when using the KK method and the F_ddPCR assay as reference test, respectively. In addition, the SjSAP4 + Sj23-LHD-ELISA (U) assay showed a greater positivity rate than the POC-CCA technique in the group of individuals with an EPG of 1–9 (43.6% vs. 16.7%, *p* = 0.0007) and in KK-negatives (49.3% vs. 6.3%, *p* < 0.0001), respectively (Table 2). The schistosomiasis prevalence determined by the urine ELISA assay was about 4-fold higher than that obtained with the immunochromatographic POC-CCA assay (48.8% vs. 12.4%, *p* < 0.0001). These observations thus indicate that the urine ELISA assay would be more effective than the POC-CCA assay in monitoring the infection transmission status in *S. japonicum*-endemic communities. Being an antibody (IgG) detection assay, the SjSAP4 + Sj23-LHD-ELISA (U) test has limited capability in discriminating a past from an active infection. However, this is potentially an important method for identifying the presence of schistosome infections in a particular population, and also to provide supporting evidence to confirm a positive case of clinical schistosomiasis.

The SjSAP4 + Sj23-LHD-ELISA (U) assay showed slight concordance with the SjSAP4 + Sj23-LHD-ELISA (S), Sj23-LHD-ELISA (S), POC-CCA, and F_ddPCR, while no significant agreement was evident with the other tests. The relatively low agreement we report is most likely due to the specific sample types used for each assay; e.g., the SjSAP4 + Sj23-LHD-ELISA (U) detects host urinary IgG, while ddPCR assays capture the parasite DNA in multiple clinical sample types, and the POC-CCA detects parasite-derived CCA in urine. Moreover, these differences can be potentiated by the fact that antibodies are detectable even in the absence of active infection and no eggs passed in the host stools. Nonetheless, the low agreement with other diagnostic tests has also been observed with other urine-based assays, including the POC-CCA (Cai et al., 2021) and U_ddPCR assay (Supplementary Table S1). In addition, it has been suggested that the enhanced leakage of anti-parasite antibodies from plasma into urine is associated with the presence of immune complexes in the kidneys as well as vascular inflammation due to glomerulonephritis (Eamudomkarn et al., 2018). In this context, the pathogenesis of glomerular disease associated with schistosomiasis is complicated, with multiple potential mechanisms such as polyclonal B-cell activation, autoimmunity, portal-systemic shunting, hepatic macrophage function, and genetic and environmental factors (chronic salmonellosis) being involved in the development of schistosomal glomerulopathy (van Velthuysen and Florquin, 2000). Therefore, in light of the above-mentioned findings, clinical urine samples are far too intricate and may contain variables that may interfere with antibody detection due to individual variability in schistosomal glomerulopathy. In addition, highly diluted urine due to water intake is among the most commonly observed factors affecting the validity of urinalysis (Franz et al., 2019). In the future, it may be necessary to collect water ingestion controlled urine samples in order to help decrease the daily fluctuation of concentrations of schistosome-derived substances (such as cell-free DNA and antigens) and specific anti-schistosome antibodies in urine samples, thus potentially increasing agreement with other diagnostic tests for urine-based assays. Another issue needs to be considered is that the influence of the storage condition of urine samples on the performance of urine-based diagnostic tests. For example, it has been reported that compared with fresh urine samples, using frozen urine (−20°C) after 1 year in the POC-CCA test resulted in a significant decrease in both the positive rate (Mewamba et al., 2021) and specificity (Graeff-Teixeira et al., 2021). Also, a previous study showed that the IgG level in urine sample without preservatives can drop rapidly upon storage at −20°C (Tencer et al., 1994). The urine samples used in this study were stored at −80°C for a long period without additives, whether such storage condition has affected the sensitivity of the SjSAP4 + Sj23-LHD-ELISA (U) assay needs to be further investigated. In addition, as high prevalence of helminth parasites other than *S. japonicum* was observed in the Palapag and Laoang endemic areas, i.e., 70.3% of study participants harbored at least two and up to five different helminth parasite species (Gordon et al., 2020), further studies on the potential antigenic cross-reactivity, especially for Sj23-LHD, are required.

## Conclusion

The SjSAP4 + Sj23-LHD-ELISA (U) assay showed a sensitivity of 47.2% and a specificity of 93.8% in the detection of low-intensity *S. japonicum* infection in KK-positive individuals from moderately endemic area. A comprehensive comparison of the performance further revealed that the urine ELISA assay was more sensitive than the SL_ddPCR test and the POC-CCA assay and had a comparable diagnostic capability with that of the U_ddPCR. The schistosomiasis prevalence determined by the urine ELISA assay was similar with that determined by the U_ddPCR, and was about 1.8-fold higher than that obtained with the KK procedure (6 slides from two stool samples). However, the urine ELISA assay provided a reduced level of sensitivity compared with more accurate assays, such as the F_ddPCR, SR_ddPCR, and serum SjSAP4 + Sj23-LHD-ELISA assay in the detection of *S. japonicum* infection in subjects with low worm burdens in rural schistosome-endemic areas in the Philippines. This study reinforces the challenge of applying urine-based tests for the diagnosis of schistosomiasis japonica in this post-MDA era, where light-intensity infections are predominant in many endemic areas.

## Data availability statement

The original contributions presented in the study are included in the article/Supplementary material, further inquiries can be directed to the corresponding author.

## Ethics statement

The studies involving human participants were reviewed and approved by The Institutional Review Board (IRB) of the Research Institute for Tropical Medicine (RITM), Manila, the Philippines; The Human Research Ethics Committee (HREC), QIMR Berghofer Medical Research Institute (QIMRB), Brisbane, Australia. Written informed consent to participate in this study was provided by the participants’ legal guardian/next of kin.

## Author contributions

YM, PC, and DM participated in conceptualization. YM, KW, RO, and PC participated in performing experiments, formal analysis, and data visualization. RO, AR, DM, and PC provided the resources. YM, KW, and PC participated in original draft preparation. AR, DM, and PC edited and revised the manuscript. DM and PC supervised the project. All authors contributed to the article and approved the submitted version.

## Funding

This research was funded by the National Health and Medical Research Council (NHMRC) of Australia (ID: APP1160046, APP2008433, APP1098244, APP1102926, and APP1037304). DM is a NHMRC Leadership Fellow and Distinguished Scientist at QIMRB. The funders had no role in study design, data collection and analysis, decision to publish, or preparation of the manuscript.

## Conflict of interest

The authors declare that the research was conducted in the absence of any commercial or financial relationships that could be construed as a potential conflict of interest.

## Publisher’s note

All claims expressed in this article are solely those of the authors and do not necessarily represent those of their affiliated organizations, or those of the publisher, the editors and the reviewers. Any product that may be evaluated in this article, or claim that may be made by its manufacturer, is not guaranteed or endorsed by the publisher.

## Footnotes

^1^ https://www.graphpad.com/quickcalcs/kappa1/
